# Multivalent mRNA Vaccine Elicits Broad Protection against SARS-CoV-2 Variants of Concern

**DOI:** 10.3390/vaccines12070714

**Published:** 2024-06-26

**Authors:** Monika Kumari, Kang-Hao Liang, Shih-Chieh Su, Hsiu-Ting Lin, Yu-Feng Lu, Ming-Jane Wu, Wan-Yu Chen, Han-Chung Wu

**Affiliations:** 1 Institute of Cellular and Organismic Biology, Academia Sinica, Taipei 11529, Taiwan; monika91@gate.sinica.edu.tw (M.K.); jay.su@academab.com (S.-C.S.); ting3772341@gate.sinica.edu.tw (H.-T.L.); yufeng.lyu@academab.com (Y.-F.L.); as0191326@gate.sinica.edu.tw (M.-J.W.); wanyuchen@gate.sinica.edu.tw (W.-Y.C.); 2 Biomedical Translation Research Center (BioTReC), Academia Sinica, Taipei 11529, Taiwan; g9580545@gate.sinica.edu.tw

**Keywords:** SARS-CoV-2, variants of concern, multivalent mRNA vaccines, vaccine efficacy

## Abstract

SARS-CoV-2 new waves are primarily caused by changes to the spike protein (S), which can substantially decrease the efficacy of vaccines. Therefore, we tested several multivalent mRNA-LNP vaccines, targeting the full-length S proteins of different variants, and identified an optimal combination for protection against VOCs in BALB/c mice. The tested formulations included trivalent (WT + BA.5 + XBB.1.5), pentavalent (WT + BA.5 + XBB.1.5 + BQ.1.1 + CH.1.1), and octavalent (WT + BA.5 + XBB.1.5 + BQ.1.1 + CH.1.1 + Alpha + Delta + BA.2) vaccines. Among these multivalent vaccines, the pentavalent vaccine showed superior protection for almost all tested variants. Despite this, each multivalent vaccine elicited greater broad-spectrum neutralizing antibodies than the previously evaluated bivalent vaccine (WT + BA.5). Subsequently, we redesigned the multivalent vaccine to efficiently generate neutralizing antibodies against recent VOCs, including EG.5.1. Immunization with the redesigned pentavalent vaccine (WT + EG.5.1 + XBB.1.16 + Delta + BA.5) showed moderate levels of protection against recent Omicron VOCs. Results suggest that the neutralization activity of multivalent vaccines is better than those of the tested bivalent vaccines against WT + BA.5 and WT + EG.5.1. Moreover, the pentavalent vaccine we developed may be highly useful for neutralizing new Omicron VOCs.

## 1. Introduction

In March 2020, the WHO declared that infection with SARS-CoV-2 and the resultant COVID-19 had reached pandemic levels [1]. Fortunately, the unprecedented development of vaccines within a one-year timeline led to a marked decline in the infection rate and death rate. Nevertheless, the continual appearance of new variants and mutations in the spike (S) protein has caused serious concern, as new waves of infections and deaths have struck different regions of the world [2]. As of 12 June 2024, there were 775,552,205 reported COVID-19 cases globally [3], and the circulating mix of older variants and newly evolved variants continues to cause new waves of disease [4]. The preferred type of vaccine has also varied with the emergence of new variants of concern (VOCs) in different regions around the world. Currently licensed vaccines were designed against the wild-type (WT) SARS-CoV-2 S protein, and the Moderna and Pfizer mRNA vaccines exhibit 90% efficacy against the WT strain [5]. In spite of this high efficacy, the utility of these approved vaccines has been weakened by the emergence of new variants. Booster doses may reduce mortality rates to some extent [5,6], but new vaccines are urgently needed. Recent studies have revealed that the Omicron sub-variants can fuse with other SARS-CoV-2 variants and cause co-infection, which may result in a new pandemic wave [4]. Therefore, multivalent vaccines may be preferable to monovalent vaccines, as the combined immunization against different variant strains may confer broad protection against emerging infections. A major advantage of multivalent vaccines is that a single injection may induce the generation of polyclonal antibodies against multiple variants, which is expected to provide better protection. Multivalent vaccination is not a new concept. A decade ago, the FDA approved a trivalent inactivated influenza vaccine (TIVc), which targets influenza A strains H1N1 and H3N2, as well as an influenza B virus strain [7]. The use of a lipid-nanoparticle-encapsulated mRNA (mRNA-LNP) platform allows for combined targeting of multiple variants and makes it easy to quickly produce a vaccine once a new variant sequence is known. In light of the fact that the European Medicines Agency (EMA) has noted that the administration of monovalent doses at short intervals may cause side effects, the use of multivalent booster doses may help to limit the number of consecutive doses while providing broad protection against SARS-CoV-2 variants [8]. Moreover, Corleis et al. recently demonstrated that multivalent mRNA vaccines can confer broad protection against Omicron sublineages at relatively low doses [9].

In a previous study, we designed a bivalent vaccine that shows broad protection against variants up to the Omicron BA.5 lineages. However, this bivalent vaccine cannot induce high levels of neutralizing antibodies against newer variants, including BQ.1.1 [10]. In this study, we describe the development of multivalent mRNA vaccine candidates, targeting several different strains. This strategy might help to reduce the need for repeated vaccinations by providing some level of protection from newly evolved VOCs. We postulated that multivalent vaccines designed to target S proteins from old and recent circulating strains may provide broad protection against current and future virus strains evolving from existing variants. The efficacies of trivalent, pentavalent, and octavalent vaccines in BALB/c mice were compared, side-by-side, with the efficacy of our previously developed bivalent vaccine (WT + BA.5). We found that the multivalent vaccines induced higher neutralizing antibody titers than the bivalent vaccine. Among the tested multivalent vaccines, the pentavalent vaccine (WT + EG.5.1 + XBB.1.16 + Delta + BA.5) provided the best broad protection against new and old virus strains.

## 2. Materials and Methods

### 2.1. Production of Modified mRNAs via In Vitro Transcription (IVT)

The WT, Alpha, Delta, BA.2, BA.5, BQ.1.1, XBB.1.5, XBB.1.16, CH.1.1, and EG.5.1 SARS-CoV-2 S cDNA plasmids were kindly provided by Dr. Yu-Chi Chou, National RNAi Core Facility, Academia Sinica, Taiwan. In brief, synthesized SARS-CoV-2 S protein-encoding DNA fragments were purchased from Integrated DNA Technologies and subcloned into pcDNA3.1 (+) expression plasmid using a GenBuilder Cloning Kit (GenScript, Piscataway, NJ, USA). 

The DNA templates used in IVT reactions were kindly provided by Dr. Mi-Hua Tao, Academia Sinica. Briefly, DNA templates were constructed to contain a T7 promoter site, one codon-optimized SARS-CoV-2 S protein (among WT, Alpha, Delta, BA.2, BA.5, BQ.1.1, XBB.1.5, XBB.1.16, and CH.1.1), a 5’ UTR, IgG kappa leader sequence, a poly(A) tail region, and the alpha-globin gene 3’ UTR. Prior to the IVT reaction, the plasmid was linearized using EcoRV and purified with the NucleoSpin Gel and PCR Clean-up Kit (Macherey and Nagel Co., Düren, Germany). The mRNA was synthesized according to the manufacturer’s recommendations using HiScribe T7 (NEB, Beverly, MA, USA) with co-transcriptional CleanCap^®^ AG (Trilink, San Diego, CA, USA) and N1-methyl-pseudouridine (Trilink, San Diego, CA, USA). Synthesized mRNA was purified via DNase I (NEB, Beverly, MA, USA) digestion, followed by LiCl (Invitrogen, Thermo Fisher Scientific, Waltham, MA, USA) precipitation and a 70% ethanol wash. Cellulose-based purification was performed to remove dsRNA from the transcribed mRNA. The final product was stored at −80 °C.

### 2.2. Preparation of mRNA-LNPs

LNP formulations were prepared using a previously described method [11]. Briefly, four types of lipids were solubilized in ethanol: SM-102 (MedChemExpress, Princeton, NJ, USA), DSPC (Avanti Polar Lipids, Alabaster, AL, USA), cholesterol (Sigma, Burlington, MA, USA), and DMG-PEG 2000 (MedChemExpress, Princeton, NJ, USA). The lipids were then mixed at a respective molar ratio of 50:10:38.5:1.5. The lipid mixture was combined with an aqueous sodium acetate buffer (25 mM, pH 4.5) containing mRNA at a flow rate ratio of 1:3 using NanoAssemblr^®^ IGNITE NxGen Cartridges (Precision NanoSystems Inc., Vancouver, BC, Canada). LNP-encapsulated mRNA samples were dialyzed against PBS (pH 7.4) at 4 °C. Then, the mRNA-LNPs were concentrated using Amicon Ultra Centrifugal Filters (10 K MWCO; Millipore, Burlington, MA, USA) and passed through a 0.45-µm filter.

### 2.3. Characterization of mRNA-LNPs

The particle size distribution, polydispersity index (PDI) value, and zeta potential of each SARS-CoV-2 S protein mRNA-LNP were analyzed via dynamic light scattering (DLS, Zetasizer Nano ZS, Malvern Instruments, Malvern, UK). Each sample was diluted 100-fold and equilibrated to 120 s at 25 °C prior to size and zeta potential measurements. The hydrodynamic diameter (z-average) and zeta potential of mRNA-LNP were analyzed by Zetasizer software, version 7.11 (www.malvern.com) (accessed on 23 June 2024). The morphology of mRNA-LNP was observed in a dry state using cryogenic transmission electron microscopy (cryo-TEM, Tecnai F20, Philips, Eindhoven, The Netherlands). Briefly, the sample solution was diluted 10-fold and transferred onto a 300-mesh copper grid covered with porous carbon film (HC300-Cu, PELCO, Fresno, CA, USA) before blotting and plunging in a 100% humidity temperature-controlled chamber from Vitrobot (FEI, Hillsboro, OR, USA). The copper grids were stored under liquid nitrogen and transferred to the electron microscope on a cryo-stage for imaging. The mRNA encapsulation efficiency (EE%) and the concentration were determined with a Quant-iT RiboGreen RNA assay kit (Invitrogen, Thermo Fisher Scientific, Waltham, MA, USA). The mRNA integrity of free mRNA and mRNA-LNP was analyzed via an agarose gel retardation assay. The mRNA-LNP complexes were solubilized with 1% Triton X-100, and the integrity of released mRNA was inspected on an agarose gel. 

### 2.4. Evaluation of In Vitro SARS-CoV-2 S Protein Expression by Flow Cytometry

SARS-CoV-2 S protein mRNA-LNPs (WT, Alpha, Delta, BA.2, BA.5, BQ.1.1, XBB.1.5, XBB.1.16, CH.1.1, and EG.5.1) were individually transfected into HEK293T cells and cultured at 37 °C in DMEM medium containing 10% FBS for 24 h. Then, the cells were collected and centrifuged. The cell pellet was washed with PBS via centrifugation, followed by incubation with fixation and permeabilization solution (BD, San Jose, CA, USA, catalog no. 554714) for 20 min at 4 °C. Cells were then stained for 1 h at 4 °C with 1 μg/mL of a monoclonal antibody specific for the RBD domain of each S protein (developed in our lab: K-RBD-mAb-75 for WT, Alpha, Delta, and BA.2 mRNA-LNP-treated cells; mRBD-8 antibody for BQ.1.1 and BA.5 mRNA-LNP-treated cells; O-RBD-mAb-11 antibody for CH.1.1 mRNA-LNP-treated cells). This step was followed by incubation with PE-conjugated goat anti-mouse IgG (1:500) for 1 h at 4 °C. The fluorescence signals were detected via a flow cytometer; a minimum of 1 × 10^4^ events were recorded for each sample and analyzed with FlowJo software (TreeStar, Ashland, OR, USA).

### 2.5. Mouse Immunization

All procedures involving animal studies were approved and performed in accordance with guidelines set by the Institutional Animal Care and Use Committee (IACUC) at Academia Sinica, Taiwan. Groups of 6- to 8-week-old female BALB/c mice were immunized via intramuscular injection with a total of 20 μg indicated SARS-CoV-2 S protein mRNA-LNP or control solution (saline) at weeks −2 and 0. Serum samples were collected 2 weeks after the second immunization and stored at −80 °C until further use.

### 2.6. Analysis of Binding Affinity of Anti-SARS-CoV-2 Antibodies from Immunized Mouse Sera

Measurements of antibody binding to various SARS-CoV-2 strains were performed using sera from immunized mice, as previously described [12]. Briefly, ELISA plates were coated with 0.5 μg/mL of variant S proteins purchased from ACRO Biosystems (Newark, DE, USA) (WT strain, SPN-C52H7; Alpha strain, SPN-C52H6; Delta strain, SPN-C52He; Omicron BA.2, SPN-C522b; BA.5, SPN-C522e; BQ.1.1, SPN-C5224; XBB.1.5, SPN-C524i, CH.1.1, SPN-C5249) and incubated at 4 °C overnight. The coated plates were blocked with 1% bovine serum albumin (BSA)/PBS at RT for 2 h. After blocking, the wells were washed twice with PBS. Then, sera that were serially diluted in 1% BSA/PBS were added to each well in triplicate, and the plate was incubated at room temperature for 1 h. After the incubation period, the plates were washed three times with PBS containing 0.1% Tween-20 (PBST_0.1_) and then incubated for 1 h with peroxidase AffiniPure goat anti-mouse IgG (H+L) (Jackson ImmunoResearch) (1:5000 dilution). After three washes with PBST_0.1_, signal was produced using 3,3′5,5′-tetramethylbenzidine (TMB) substrate (TMBW-1000-01, SURMODICS). Finally, the reaction was stopped with 3 N HCl, and absorbance was measured at 450 nm by an ELISA reader (Versa Max Tunable Microplate Reader; Molecular Devices, Sunnyvale, CA, USA).

### 2.7. Pseudovirus Neutralization Assay

Blood samples of vaccinated mice were collected 4 weeks after the first booster shot, and sera were isolated to determine the neutralization activity against different SARS-CoV-2 pseudoviruses. The pseudovirus neutralization assays were performed using SARS-CoV-2 pseudotyped lentiviruses expressing full-length S protein and firefly luciferase. The lentiviruses were used to transduce HEK293T cells that overexpressed human ACE2 (HEK293T/hACE2; purchased from the National RNAi Core Facility, Academia Sinica, Taiwan). The serum from each mouse was serially diluted using 1% FBS DMEM and pre-incubated with 1000 TU SARS-CoV-2 pseudovirus for 1 h at 37 °C. After incubation, the mixtures were added to 1 × 10^4^ HEK293T/hACE2 cells, which had been pre-seeded into each well of a 96-well white plate (Thermo Fisher Scientific, Waltham, MA, USA) for 24 h at 37 °C. The pseudovirus-containing culture medium was then replaced with 10% FBS DMEM for an additional 48 h incubation. Next, ONE-Glo luciferase reagent (Promega, Madison, WA, USA) was added to each well for 3 min incubation at 37 °C to measure firefly luciferase activity. Luminescence was measured using a microplate spectrophotometer (Molecular Devices, Sunnyvale, CA, USA) to determine pseudovirus infection efficacy. The half-maximal inhibitory concentration (IC_50_) was calculated via nonlinear regression using Prism software version 8.1.0 (GraphPad Software Inc., San Diego, CA, USA). The average IC_50_ value for each experimental group was determined from three independent experiments.

### 2.8. Statistical Analyses

All data are expressed as mean ± SEM from at least three independent experiments. Significant differences were calculated using the Student’s *t*-test. *p*-values less than 0.05 were considered significant. * *p* < 0.05, ** *p* < 0.01, *** *p* < 0.001.

## 3. Results

Using a similar approach, researchers are currently working to overcome the diminished effects of licensed vaccines against different SARS-CoV-2 VOCs by generating multivalent vaccines that target receptor-binding domain (RBD) sequences of S protein from multiple SARS-CoV-2 VOCs. Along the same lines, we generated three bivalent vaccines targeting S proteins from BA.5 + WT, BA.5 + Alpha, and BA.5 + Delta, and we performed a side-by-side comparison of the efficacies of these three vaccines. While all three bivalent vaccines protect better than monovalent vaccines, the efficacies wane against emerging variants such as BQ.1.1 [10]. Therefore, in this study, we designed multivalent vaccines toward different strains and again compared the efficacies of these vaccines side-by-side to identify the most effective multivalent vaccine.

**Figure 1:**
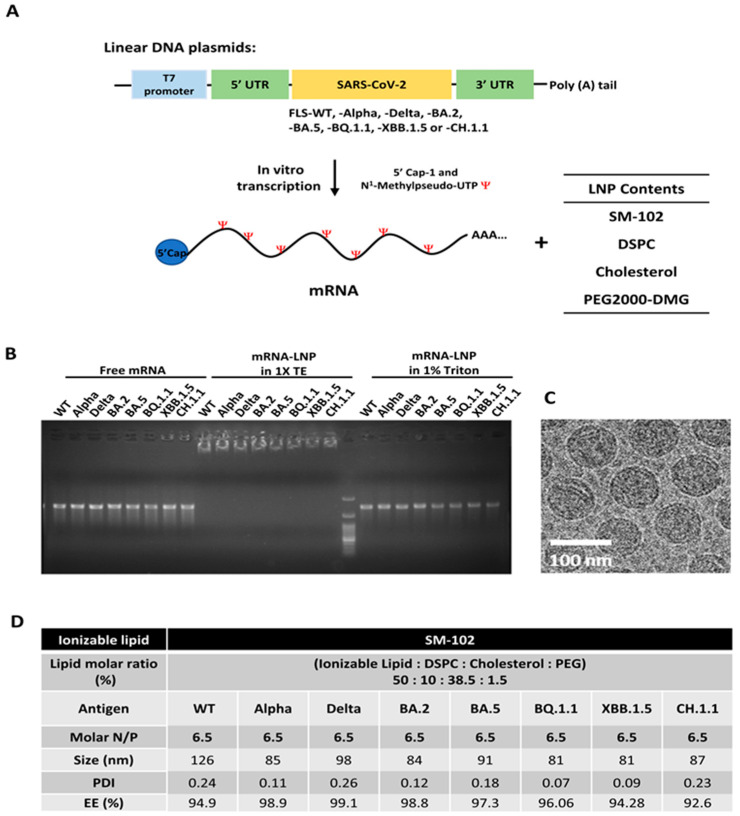
Characterization of mRNAs and mRNA-LNPs. (**A**) Schematic diagram illustrates the synthesis of different mRNAs encoding SARS-CoV-2 S protein variants. (**B**) Gel electrophoresis assay. mRNA-LNPs were incubated in 1X TE buffer and 1% Triton. Free mRNAs served as negative controls. The 100 bp DNA ladder from ArrowTech life science was used as a molecular weight marker. (**C**) Cryo-TEM images illustrate the structures of mRNA-LNPs. (**D**) Summary table shows the molar ratio and physiochemical properties of the mRNA-LNPs.

We first generated mRNAs encoding full-length S proteins from WT, Alpha, Delta, BA.2, BA.5, BQ.1.1, XBB.1.5, CH.1.1, XBB.1.16, and EG.5.1 SARS-CoV-2 strains. The mRNA sequences were modified with pseudouridine, and the coding sequences were flanked by 5′ and 3′ UTR regions from the human hemoglobin subunit alpha 1 mRNA to regulate mRNA stability and protein expression (Figure 1A). Agarose gel electrophoresis was used to assess the quality of synthesized mRNAs (Figure 1B). To prepare mRNA-LNP complexes with the mRNAs, an ethanol solution of lipid mixture was combined with an aqueous solution of mRNAs in a NanoAssemblr^®^ Ignite microfluidic mixing device.

### 3.1. Physiochemical Characterization of mRNA-LNP Complexes

The quality of packed mRNA is analyzed by using agarose gel retardation assay (Figure 1B). The mRNA-LNP diluted with 1× TE buffer shows no trace of free mRNA, and the complex remains trapped in the well. However, disrupting the LNP with 1% Triton X-100 detergent releases mRNA from complex, and bands can be observed at a similar molecular weight as the free mRNA control.

The average particle sizes and polydispersity index (PDI) values of the mRNA-LNP complexes were measured using dynamic light scattering (DLS). The sizes were in the range of 80–130 nm, which is suitable for nanoparticle endocytosis. The PDI values for all mRNA-LNP complexes were less than 0.3 (Figure 1D). Thus, irrespective of the encapsulated mRNA, uniformity of mRNA-LNP complexes was maintained. Cryo-EM analysis further revealed that the mRNA-LNP complexes were spherical, and the particles were uniformly distributed in solution without aggregation (Figure 1C).

### 3.2. Validation of Protein Expression In Vitro

**Figure 2:**
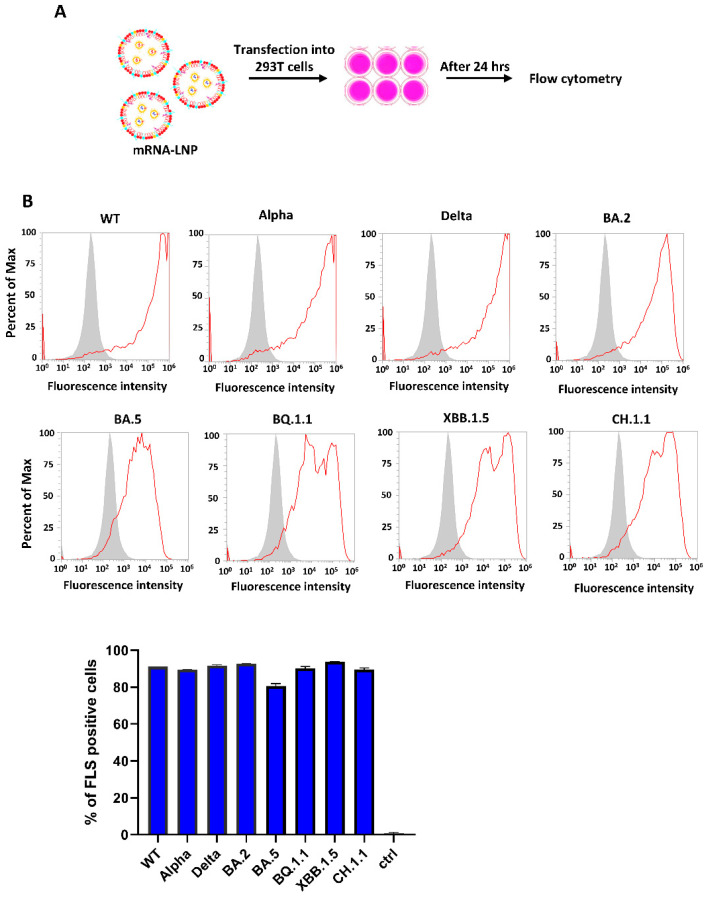
293T cells treated with different mRNA-LNPs express S proteins. (**A**) Schematic diagram illustrates the cell-based antigen expression assay. (**B**) Flow cytometry data show the successful transfection and expression of each mRNA. Quantitative flow cytometry data are shown in the bar graph. All experiments were performed in triplicate; standard deviations are shown as error bars. The control group included non-treated cells and had no specific binding activity.

The functionality of each mRNA was confirmed by a cell-based assay. The mRNA-LNP complexes containing respective mRNAs were applied to 293T cells, as shown in Figure 2A. After 24 h of incubation, the treated cells were collected in dissociation buffer and incubated with a fluorescently labeled antibody against S protein. WT, Alpha, Delta, and BA.2 mRNA-LNP-treated cells were incubated with the k-RBD-chAb-75 antibody developed in our lab [13]. BQ.1.1 and BA.5 mRNA-LNP-treated cells were incubated with mRBD-8 antibody. CH.1.1 mRNA-LNP-treated cells were incubated with O-RBD-mAb-11 antibody. All of these antibodies were developed in our lab and were chosen based on their binding activities toward S proteins of different variants. The successful binding of cells with the respective antibodies resulted in a shifted peak with respect to non-treated cells when fluorescence was measured via flow cytometry (Figure 2B). Quantitative analysis of the flow cytometry data allowed us to directly measure the proportions of cells expressing full-length S proteins. 

### 3.3. Validation of Antibody Production from Immunized Mice Sera

**Figure 3:**
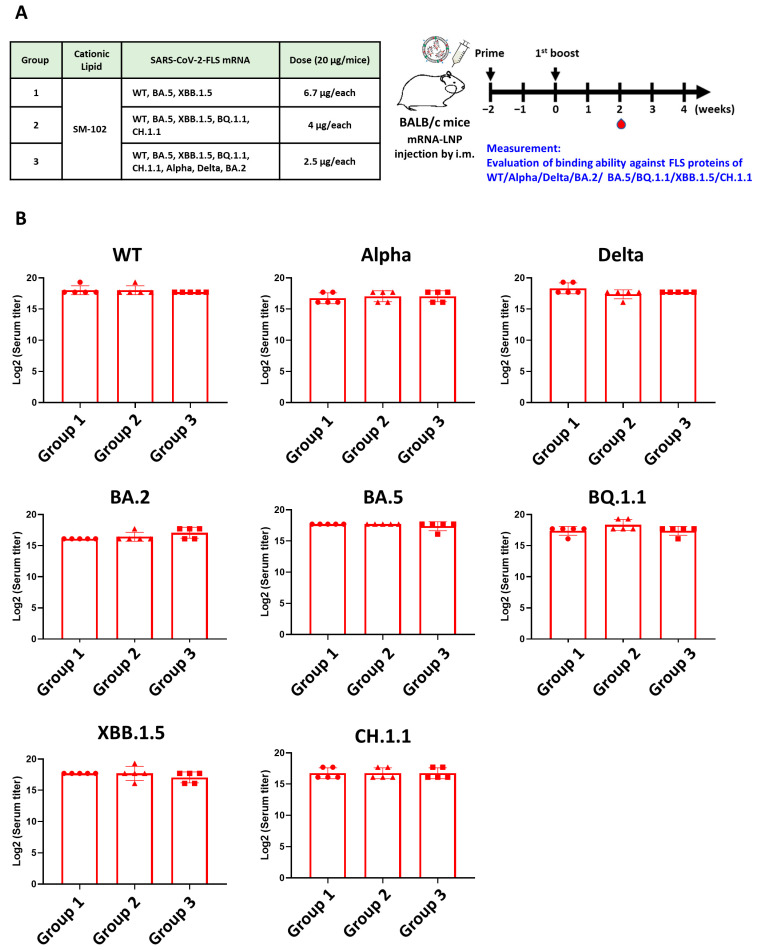
Serum binding activities against different strains by ELISA. (**A**) Schematic illustration shows immunization strategy (*n* = 5 per group). (**B**) The binding activities of immunized sera against S protein variants were evaluated. All experiments were performed in triplicate; standard deviations are shown as error bars. Log2 (serum titer) indicates the limit of detection for binding of the indicated S protein by each diluted serum sample. Different symbols represent different groups.

Since a number of variants are rapidly evolving around the world, the effectiveness of monovalent and bivalent vaccines has become limited. Therefore, we combined recently emerging variants with WT + BA.5 variants, and we analyzed the binding efficacy and neutralizing titers against newly emerging variants BQ.1.1, XBB.1.5, and CH.1.1. We anticipated that the multivalent vaccines would induce varied antibody responses against multiple S proteins, giving rise to a polyclonal binding profile that can enhance the neutralizing titers against most variants. The three immunized groups are shown in Figure 3A. Group 1 received a trivalent combination of WT + BA.5 + XBB.1.5. Group 2 received a pentavalent combination of WT + BA.5 + XBB.1.5 + BQ.1.1 + CH.1.1. Group 3 received an octavalent combination of WT + BA.5 + XBB.1.5 + BQ.1.1 + CH.1.1 + Alpha + Delta + BA.2. Each group received a total of 20 μg of mRNA, which was divided equally among the variants to ensure that a minimum of 2.5 μg of respective mRNA variant was present in the octavalent vaccine. Nevertheless, serum binding activities against different variants showed no significant differences between vaccination groups (Figure 3B).

### 3.4. Multivalent mRNA Vaccination Induces Cross-Variant Neutralization of SARS-CoV-2 Variant Pseudoviruses

**Figure 4:**
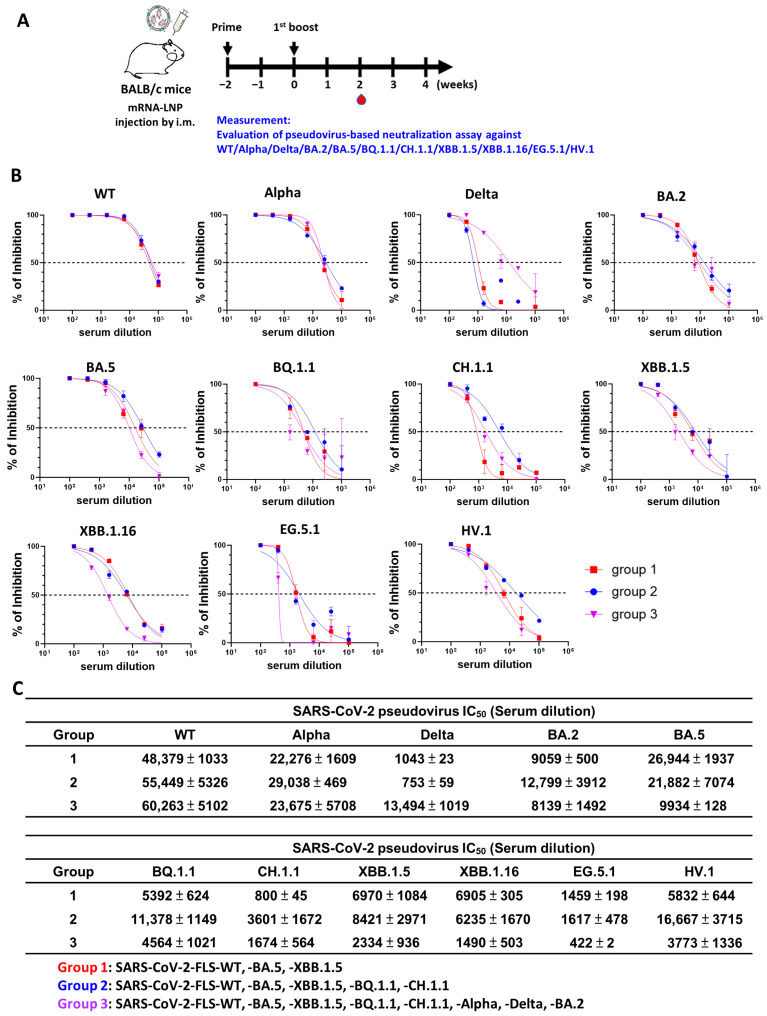
Multivalent mRNA vaccination induces neutralizing activity against SARS-CoV-2 variant pseudoviruses. (**A**) Mouse model immunization and blood sample collection schedules (*n* = 5 per group). (**B**) Pseudovirus neutralization assay was performed on sera collected from mice receiving multivalent mRNA vaccination against wild-type (WT), Alpha, Delta, BA.2, BA.5, BQ.1.1, CH.1.1, XBB.1.5, XBB.1.16, EG.5.1, and HV.1. (**C**) Half-maximal inhibitory concentrations (IC_50_ values) for sera of multivalent-mRNA-vaccinated mice tested against SARS-CoV-2 variant pseudoviruses. Results are representative of three independent experiments performed in triplicate; standard deviations are shown as error bars.

Sera from vaccinated mice were tested for the capacity to neutralize pseudoviruses. Figure 4A shows the immunization schedule. The group 1 mice immunized with trivalent mRNA-LNP vaccine (WT + BA.5 + XBB.1.5) showed high levels of protection against WT, Alpha and BA.5, moderate levels of protection against BA.2, B.Q.1.1, XBB.1.5, XBB.1.16, and HV.1, and minimal protection against Delta, EG.5.1, and CH.1.1. Group 2 mice immunized with the pentavalent mRNA-LNP vaccine (WT + BA.5 + XBB.1.5 + BQ.1.1 + CH.1.1) showed high levels of protection against WT, Alpha, BA.2, BA.5, B.Q.1.1, and HV.1, moderate protection against CH.1.1, XBB.1.5, XBB.1.16, and EG.5.1, and minimal protection against Delta. Group 3 mice receiving the octavalent mRNA-LNP vaccine (WT + BA.5 + XBB.1.5 + BQ.1.1 + CH.1.1 + Alpha + Delta + BA.2) exhibited high levels of protection against WT, Alpha, and Delta, and moderate protection against BA.2, BA.5, B.Q.1.1, CH.1.1, XBB.1.5, XBB.1.16, and HV.1 (Figure 4B,C). The neutralization titers (IC_50_) were graphed as the serum dilution showing 50% neutralization of the tested pseudovirus (Figure S1). According to our assays on sera from mice immunized with a bivalent vaccine (WT + BA.5), the bivalent was not effective against emerging variants like BQ.1.1, CH.1.1, XBB.1.5, XBB.1.16, and EG.5.1 (Figure S2). Nevertheless, the bivalent vaccine did provide generally broader protection than monovalent vaccines. Importantly, sera from mice immunized with trivalent vaccines targeting WT, BA.5, and XBB.1.5 exhibited better neutralizing activities against newly emerging variants compared to those receiving bivalent vaccines (Figure S1). Sera from mice immunized with the pentavalent vaccine showed high levels of protection against most of the new emerging variants (Figure S1). Interestingly, sera from the trivalent- or octavalent-vaccine-immunized mice showed a lower level of protection against most of the tested variants than that from the pentavalent-vaccine-immunized mice.

**Figure 5:**
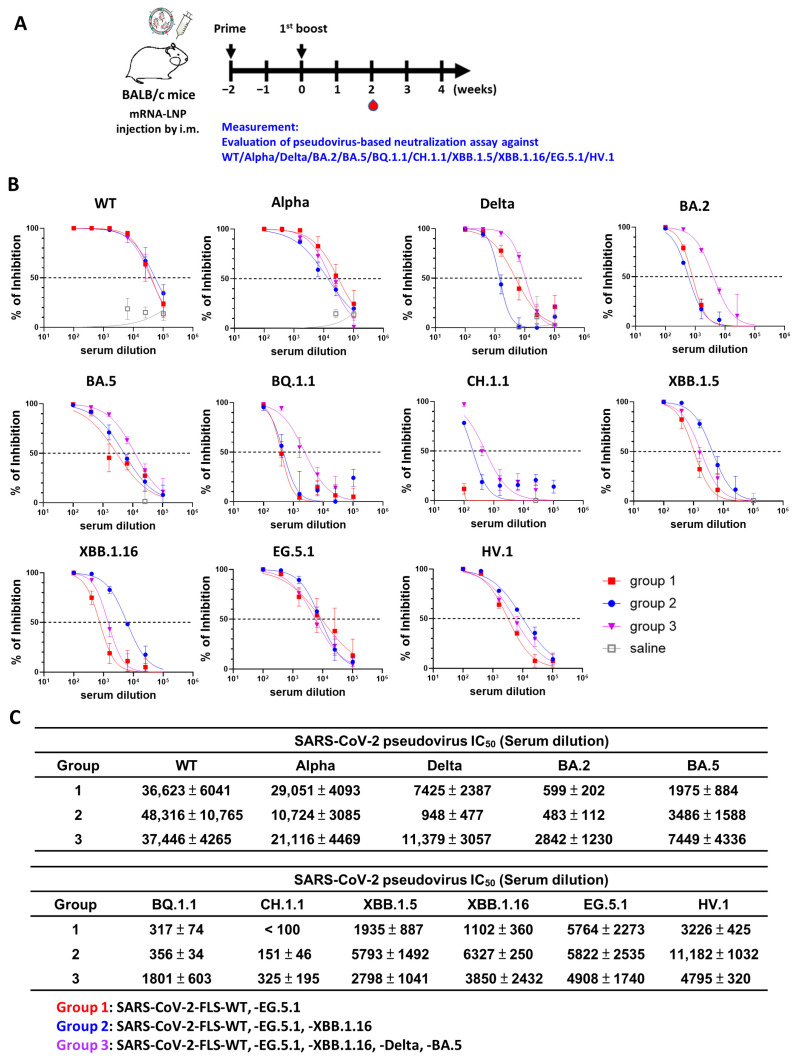
Redesigned multivalent mRNA vaccination induces neutralizing activity against SARS-CoV-2 variant pseudoviruses. (**A**) Mouse model immunization and blood sample collection schedules (*n* = 5 per group). (**B**) Pseudovirus neutralization assay was performed on sera collected from mice receiving the indicated multivalent mRNA vaccinations. Neutralization was tested for wild-type (WT), Alpha, Delta, BA.2, BA.5, BQ.1.1, CH.1.1, XBB.1.5, XBB.1.16, EG.5.1, and HV.1. (**C**) Half-maximal inhibitory concentrations (IC_50_ values) for sera of multivalent-mRNA-vaccinated mice, tested against SARS-CoV-2 variant pseudoviruses. Results are representative of three independent experiments performed in triplicate; standard deviations are shown as error bars.

After confirming the potential of multivalent vaccines to induce neutralizing responses, the design strategy was adjusted to more precisely target some of the most concerning emerging variants (Figure 5). The tested formulations included a bivalent (WT + EG.5.1), trivalent (WT + EG.5.1 + XBB.1.16), and pentavalent (WT + EG.5.1 + XBB.1.16 + Delta + BA.5) vaccine. The bivalent vaccine (WT + EG.5.1) showed high protection against WT and Alpha, with moderate protection against Delta, BA.5, XBB.1.5, XBB.1.16, EG.5.1, and HV.1, and low protection against BA.2, BQ.1.1, and CH.1.1. The trivalent vaccine (WT + EG.5.1 + XBB.1.16) showed high protection against WT, Alpha, and HV.1, with moderate protection against BA.5, XBB.1.5, XBB.1.16, and EG.5.1, and low protection against Delta, BA.2, BQ.1.1, and CH.1.1. The pentavalent vaccine (WT + EG.5.1 + XBB.1.16 + Delta + BA.5) showed high protection against WT, Alpha, and Delta, with moderate protection against BA.2, BA.5, BQ.1.1, XBB.1.5, XBB.1.16, EG.5.1, and HV.1, and low protection against CH.1.1. Notably, the pentavalent vaccine targeted a combination of old and new variants, and it showed the broadest range of neutralization against the tested variants. Interestingly, the bivalent vaccine (WT + EG.5.1) showed low protection against the parental XBB.1.5 variant. EG.5 is a descendent lineage of XBB with a F456L mutation in the spike protein. An additional Q52H mutation in the spike protein of EG.5 leads to subvariant EG.5.1 [14]. Thus, our data in Figure 5 show that the mutation of only two amino acids can substantially diminish the neutralization activity toward emerging variants. Overall, these data highlight the fact that vaccine composition is a crucial factor for protection against new emerging variants. Moreover, our comparisons of multivalent vaccines demonstrate that pentavalent vaccines can confer at least moderate levels of protection against several key variants. 

## 4. Discussion

The periodic occurrence of new mutations in the SARS-CoV-2 viral genome (especially in the S protein) has been linked to higher virus transmissibility and lower vaccine efficacy [15]. Although vaccine booster doses have proven to be an effective means of enhancing neutralizing antibody titers, this approach alone cannot sufficiently prevent disease. For instance, Walls et al. demonstrated that the individuals who received a third booster dose had high titers of a broad range of neutralizing antibodies and potent polyclonal antibodies, suggesting that repeated exposure to SARS-CoV-2 can enhance the antibody response [16]. However, mixed exposure to old and new variants still threatens to undermine monovalent vaccine efficacy in different regions of the world [4]. As such, licensed vaccines must be continually evaluated against emerging variants and mixed variants. Over time, the usage of each vaccine must be fine-tuned or discontinued based on faltering efficacies. For instance, the US FDA updated their recommended vaccine booster dose regimen to include a bivalent vaccine from Moderna (original and Omicron BA.1) and Pfizer-BioNTech (original and Omicron BA.4/BA.5), replacing the recommended use of monovalent vaccines [17]. Moreover, the US FDA, based on several pieces of evidence, updated COVID-19 vaccines for use in the fall of 2023. The US FDA has advised manufactures to develop vaccines with a monovalent XBB.1.5 composition [18]. As of now, the WHO advisory committee makes vaccine recommendations based on designated VOCs. The updated composition of a recommended vaccine is dependent on its broad protection and ability to induce cross-reactive neutralizing antibodies against VOCs [19]. In addition, there are a few studies that have shown that individuals who are naturally infected with different variants of the SARS-CoV-2 virus show broad protection against newly emerging VOCs [19,20]. We and others have further shown that bivalent vaccines may neutralize a wide range of variants, including Omicron, and are likely to be more effective than monovalent vaccines [10,21]. Although bivalent vaccines can induce higher neutralizing antibody titers than monovalent vaccines, the bivalent mRNA-LNP vaccines targeting BA.5 + WT, BA.5 + Alpha, and BA.5 + Delta still show little protection against the new Omicron sublineage BQ.1.1 [10]. Therefore, an urgent need remains for the development of new vaccines to effectively fight emerging Omicron variants. Here, we first demonstrate that multivalent mRNA-LNPs, such as trivalent, pentavalent, and octavalent vaccines, target a broad selection of VOCs and induce higher neutralizing antibody titers than the bivalent (WT + BA.5) control. 

Bivalent vaccines can induce more comprehensive protection than monovalent vaccines, but they may not readily confer the ability to neutralize newly emerging variants. An immunogenicity comparison of two different bivalent vaccine compositions was performed recently (25 μg each of the mRNA variants; comparing between WT + BA.1 and WT + BA.4/5). Both bivalent compositions induced greater neutralizing antibody activity than monovalent compositions, and among the tested bivalent vaccines, WT + BA.4/5 was slightly more effective than WT + BA.1 [22]. Thus, the development of vaccines targeting appropriate variants appears to be crucial for providing protection against new emerging diseases. Furthermore, our data support the conclusion that targeting different combinations of variants can enhance the induction of neutralizing activity against VOCs. Importantly, our data show that all tested multivalent vaccines better elicited broadly neutralizing antibodies against VOCs compared with bivalent vaccines.

Despite the different concentrations of each variant mRNA type between groups, we expected that the dose difference between variants in the three groups should not affect the neutralizing antibody response and that the overall mRNA dose should not affect toxicity. In a previous study, Hajnik et al. posited that doses of mRNA higher than 2 μg to 4 μg would not result in a stronger antibody responses against SARS-CoV-2 strains [23]. Similarly, a comparison of immunogenic responses in BALB/c mice immunized with a wide range of mRNA-1273 doses (0.0025–20 μg) revealed that increasing the dose of mRNA from 0.0025 to 2.5 μg significantly increased the levels of neutralizing and binding antibodies. However, a plateau in the levels of neutralizing and binding antibodies was seen after mice received doses ranging from 5 to 20 μg [24]. In another study, Laczko et al. demonstrated that mRNA doses of 30 μg did not induce major cytotoxicity [25]. Therefore, in this study, we chose 2.5 μg as a minimal dose for each variant in the octavalent group. 

The mRNA vaccines deployed to reduce infection and mortality rates in COVID-19 represented a breakthrough technology [2,26]. As such, mRNA vaccines now provide an alternative technology for targeting a wide range of infectious diseases, such as influenza [27]. Because there are at least 18 different influenza A virus subtypes and it is difficult to accurately predict which will rise to prominence each year, a multivalent influenza vaccine might provide more complete protection against diverse influenza virus subtypes and prevent the next influenza pandemic [28]. Along these lines, one study showed that a quadrivalent vaccine containing mRNAs encoding the hemagglutinin (HA) of influenza virus H1 and H3, in addition to the neuraminidase (NA) of influenza virus N1 and N2, could be efficiently delivered, and it causes expression of all four antigens; the expressed antigens were sufficiently immunogenic and induced a strong functional antibody response to provide protection against viral challenge in mice [29]. Arevalo et al. further used mRNA-LNP technology to develop a multivalent vaccine encoding the HA antigens of all 20 known influenza A and B virus subtypes. This multivalent vaccine elicited high levels of cross-reactive and subtype-specific antibodies, and it protected mice and ferrets from matched and mismatched influenza virus strains [30]. Moreover, a quadrivalent mRNA vaccine targeting HA, NA, matrix protein 2, and nucleoprotein of the influenza A group 2 viruses could induce broadly cross-reactive immune responses, and this vaccine provided broad protection in mice from all tested virus challenges, including challenge with a relevant H1N1 influenza virus group 1 strain [31]. In addition to influenza vaccines, several mRNA vaccines targeting multiple infectious diseases are currently in clinical development. These include vaccines for Dengue [32], Zika [33], Chikungunya [34], Ebola [35], and HIV [36]. Taken together, these studies reveal the great potential and importance of mRNA-LNP technology in vaccine and drug development.

Recently, Khan et al. showed that the trivalent protein subunit vaccine against B.1.1.7, B.1.351, and P.1 S protein induces a broader humoral response and antibodies that inhibit ACE2 binding, as compared with the monovalent vaccine [37]. Along the same lines, we developed trivalent, pentavalent, and octavalent vaccines composed of different variants and performed a side-by-side comparison of bivalent and combinational mRNA-LNP vaccine efficacies against WT, Alpha, Delta, BA.2, BA.5, BQ.1.1, CH.1.1, XBB.1.5, XBB.1.16, EG.5.1, and HV.1. We chose to focus on the level of neutralizing antibody induction because it plays a major role in protecting an individual from WT and variant SARS-CoV-2 virus infections. We demonstrated that the individual mRNA doses included in all three tested multivalent vaccines are sufficient to induce similar levels of neutralizing antibodies against all tested variants. Even the lowest individual mRNA doses (in the octavalent vaccine) did not induce lower serum binding levels. This result suggests that immunization against multiple variants at lower individual doses may not drastically affect antibody production and binding efficiency. Moreover, our detailed investigation of neutralizing antibodies in immunized mouse sera showed that the trivalent, pentavalent, and octavalent vaccines were more effective than the bivalent vaccine. Among all tested vaccines, the pentavalent mixture showed the strongest neutralization against new emerging variants, such as BQ.1.1, CH.1.1, XBB.1.5, and XBB.1.16, and low neutralization against EG.5.1. The data showed that a mutation in a single amino acid (e.g., from XBB lineage to EG.5) can decrease the neutralization activity of the existing vaccine. Therefore, we redesigned the vaccine composition and screened for high neutralization titer against the emerging variant EG.5.1. Inclusion of EG.5.1 as a targeted variant increased the neutralization activity against EG.5.1 and also did not disrupt the neutralization of parental lineages, such as XBB. Moreover, the combination vaccine targeting WT + EG.5.1 produced no neutralization against BA.2, BQ.1.1, and CH.1.1. The co-circulation of different variants around the world creates a need for vaccines that can potentially reduce the viral infection burden of multiple known VOCs. Otherwise, the virus will continue to mutate and may generate new variants that cause more serious infection. As such, we sequentially designed multivalent vaccines that are suitable to neutralize many of the known VOCs. We expect this information will be helpful for researchers seeking to improve the design of vaccines. In particular, our data indicate that the redesigned pentavalent (WT + EG.5.1 + XBB.1.16 + Delta + BA.5) vaccine showed broad neutralization and was effective against almost all the tested variants, possibly due to the fact that this pentavalent vaccine consists of SARS-CoV-2 original variants and new spread variants from different lineages. 

## 5. Conclusions

The purposes of this study were to test whether a multivalent vaccine is more effective than bivalent vaccines and to identify the best combination of targeted variants to create an effective vaccine against WT and emerging VOCs. We have performed a side-by-side comparison of different mRNA-LNP vaccines’ composition in terms of their efficacies against WT, Alpha, Delta, BA.2, BA.5, BQ.1.1, CH.1.1, XBB.1.5, XBB.1.16, EG.5.1, and HV.1. The data indicate that the multivalent vaccines show better neutralization activity than the bivalent vaccine (WT + BA.5). Moreover, we found a pentavalent vaccine (WT + EG.5.1 + XBB.1.16 + Delta + BA.5) that induced an excellent neutralization response against new Omicron VOCs. The results of this study suggest that a combination vaccine may help us to overcome the waning efficacy of current products and prevent future pandemics.

## Data Availability

The datasets analyzed in the current study are available upon reasonable request.

## Supplementary Materials

The following supporting information can be downloaded at: https://www.mdpi.com/article/10.3390/vaccines12070714/s1, Figure S1: This graph shows the IC_50_ titers for the three vaccination groups sera collected post-vaccination to neutralize respective pseudoviruses; Figure S2: IC_50_ values for sera of bivalent mRNA-vaccinated mice tested against SARS-CoV-2 variant pseudoviruses.

## Abbreviations

COVID-19: Coronavirus disease 2019; SARS-CoV-2: severe acute respiratory syndrome coronavirus 2; hACE2: human angiotensin converting enzyme 2; IC_50_: half maximal inhibitory concentration; VOCs: variants of concern.
