# Supplementary material for: Multivalent mRNA Vaccine Elicits Broad Protection against SARS-CoV-2 Variants of Concern
Source: Vaccines (Basel). 2024 Jun 26;12(7):714. doi: 10.3390/vaccines12070714 (PMC11281580; doi:10.3390/vaccines12070714)

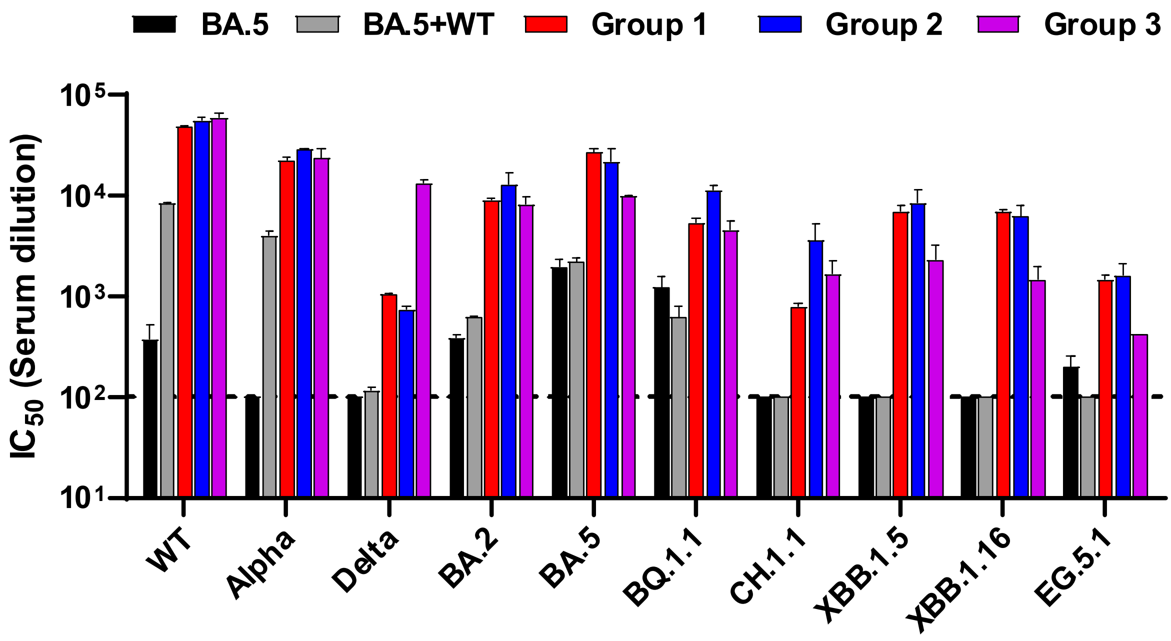

Figure S1: This graph shows the IC_{50} titers for the vaccination groups sera collected post-vaccination to neutralize respective pseudoviruses.

	SARS-CoV-2 pseudovirus IC_{50} (Serum dilution)									
Group	WT	Alpha	Delta	BA.2	BA.5	BQ.1.1	CH.1.1	XBB.1.5	XBB.1.16	EG.5.1
BA.5 (10 μg/mice)	377 ± 139	<100	<100	392 ± 22	1,984 ± 328	1,237 ± 340	<100	<100	<100	201 ± 57
WT + BA.5 (5 + 5 μg/mice)	8,334 ± 59	4,063 ± 453	115 ± 9	628 ± 2	2,216 ± 213	621 ± 169	<100	<100	<100	<100

Figure S2. IC_{50} values for sera of bivalent mRNA-vaccinated mice tested against SARS-CoV-2 variant pseudoviruses.

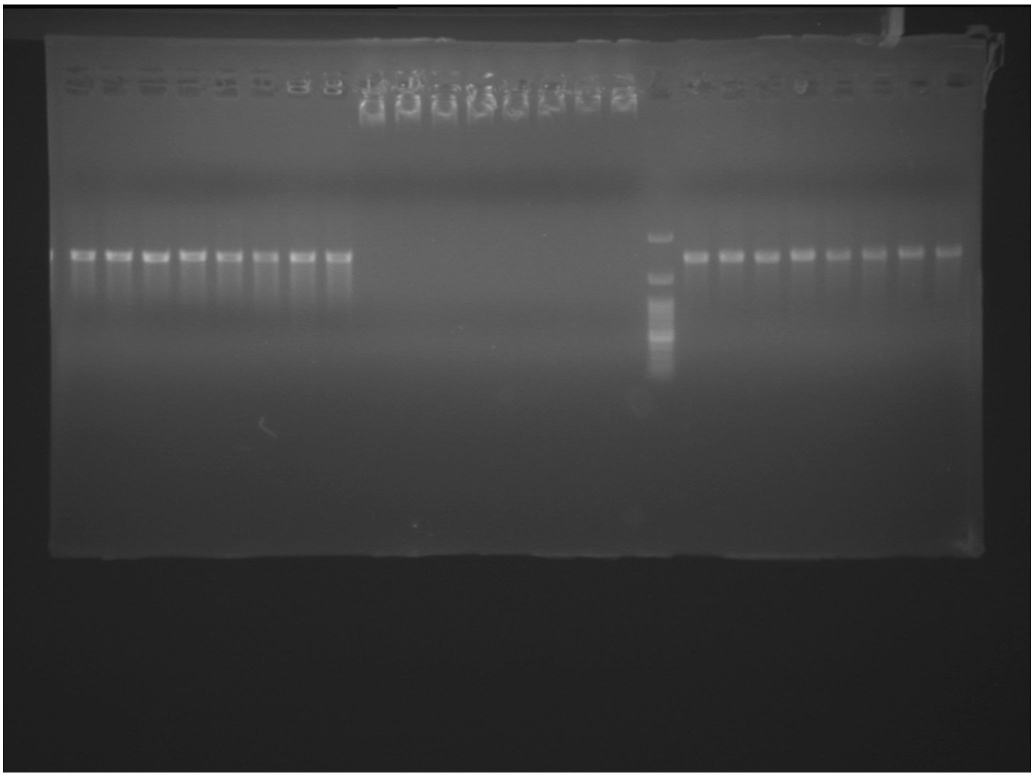

Supplement: Supplementary file 1 [file vaccines-12-00714-s001.zip › vaccines-3003549-supplementary.pdf]
